# Revealing the Hyperdiverse Mite Fauna of Subarctic Canada through DNA Barcoding

**DOI:** 10.1371/journal.pone.0048755

**Published:** 2012-11-02

**Authors:** Monica R. Young, Valerie M. Behan-Pelletier, Paul D. N. Hebert

**Affiliations:** 1 Biodiversity Institute of Ontario and Department of Integrative Biology, University of Guelph, Guelph, Ontario, Canada; 2 Agriculture and Agri-Food Canada, Ottawa, Ontario, Canada; University of Western Ontario, Canada

## Abstract

Although mites are one of the most abundant and diverse groups of arthropods, they are rarely targeted for detailed biodiversity surveys due to taxonomic constraints. We address this gap through DNA barcoding, evaluating acarine diversity at Churchill, Manitoba, a site on the tundra-taiga transition. Barcode analysis of 6279 specimens revealed nearly 900 presumptive species of mites with high species turnover between substrates and between forested and non-forested sites. Accumulation curves have not reached an asymptote for any of the three mite orders investigated, and estimates suggest that more than 1200 species of Acari occur at this locality. The coupling of DNA barcode results with taxonomic assignments revealed that Trombidiformes compose 49% of the fauna, a larger fraction than expected based on prior studies. This investigation demonstrates the efficacy of DNA barcoding in facilitating biodiversity assessments of hyperdiverse taxa.

## Introduction

Species identification and discovery has been greatly accelerated by DNA barcoding, the analysis of sequence variation in a 648 base pair segment of the mitochondrial CO1 gene [1]. DNA barcoding has been successful in many animal groups [1]–[4], reflecting the fact that intraspecific sequence variation is consistently low, typically a fraction of a percent, while interspecific divergence usually exceeds 2%. When deep intraspecific variation is detected, cryptic species are often subsequently revealed through ecological or morphological study [5], [6].

The congruence in patterns of sequence variation across different taxonomic lineages allows the use of DNA barcodes to explore biodiversity in groups which lack a well-developed taxonomic framework. It facilitates rapid diversity assessment in such cases by enabling the delineation of MOTUs, molecular operational taxonomic units [7]. Because the quantification of biodiversity is transparent and reproducible, DNA barcoding is becoming a standard practice for assessing diversity patterns in poorly known taxa [4], [8], [9].

Although only 45,000 species have been described, Acari (mites) are believed to be one of the most diverse groups of arthropods, perhaps including more than 1 million species [10]. They are certainly one of the most abundant groups of arthropods as mite densities reach nearly 2 M individuals/m^2^ in temperate deciduous forest sites [11], nearly 0.5 M/m^2^ in dry tropical forests [11], and more than 0.1 M/m^2^ in northern sites [12]. Although they are often treated as members of the soil fauna, mites are associated with varied substrates [13] forming distinct assemblages on tree trunks, in soils, in surface litter, on fungi, and in aquatic habitats [10], [13]–[15].

The three dominant orders of soil mites have varied feeding modes, genetic systems and dispersal mechanisms [16]. The Sarcoptiformes are generally mycophagous or saprophagous feeders [16] with long adult lifespans [17] and a thelytokous parthenogenetic genetic system [18]. By contrast, the Mesostigmata tend to be free-living predators or parasites [16] with short adult lifespans and haplodiploid genetic systems [18]. Members of a third order, the Trombidiformes, show the greatest diversity in feeding mode (animal and plant parasites, free living predators, free living detritivores), and in genetic systems [16], [18].

Despite their diversity and abundance, mites are rarely included in biodiversity assessments because of serious taxonomic barriers. The status of many species is uncertain due to synonymies [19], morphotypes which are distinct species [20], and sexual dimorphisms [21]. Immature life stages are also excluded from surveys as they lack diagnostic morphological characters. Aside from these challenges, there is a scarcity of taxonomic experts. Consequently, surveys are often limited to higher level taxonomic assignments [22]–[25], or to assessments of a particular group [13], [26]–[28]. These factors preclude detailed assessments of the fauna, such as the examination of species turnover in space or time. DNA barcoding has the potential to radically advance our understanding of both the extent and patterns of species diversity in mites by providing a transparent, consistent method for delineating species which allows the inclusion of all life stages and both sexes. DNA barcoding has been successfully used in delimiting mite species [29], but prior work has focused on phylogenetic studies of a few species or genera [30]–[32].

Our work assesses the diversity of the mite fauna at one site in the Canadian subarctic and determines the extent of faunal divergence between major habitats. As such, it represents the first comprehensive assessment of mite diversity using molecular methods in any geographic setting.

## Materials and Methods

### Ethics Statement

No specific permits were required for the described field studies. Additionally, no specific permissions were required for these collection locations/activities as they are not privately owned or protected, and did not involve the collection of endangered or protected species.

### Study Site/Sampling Design

**Figure 1:**
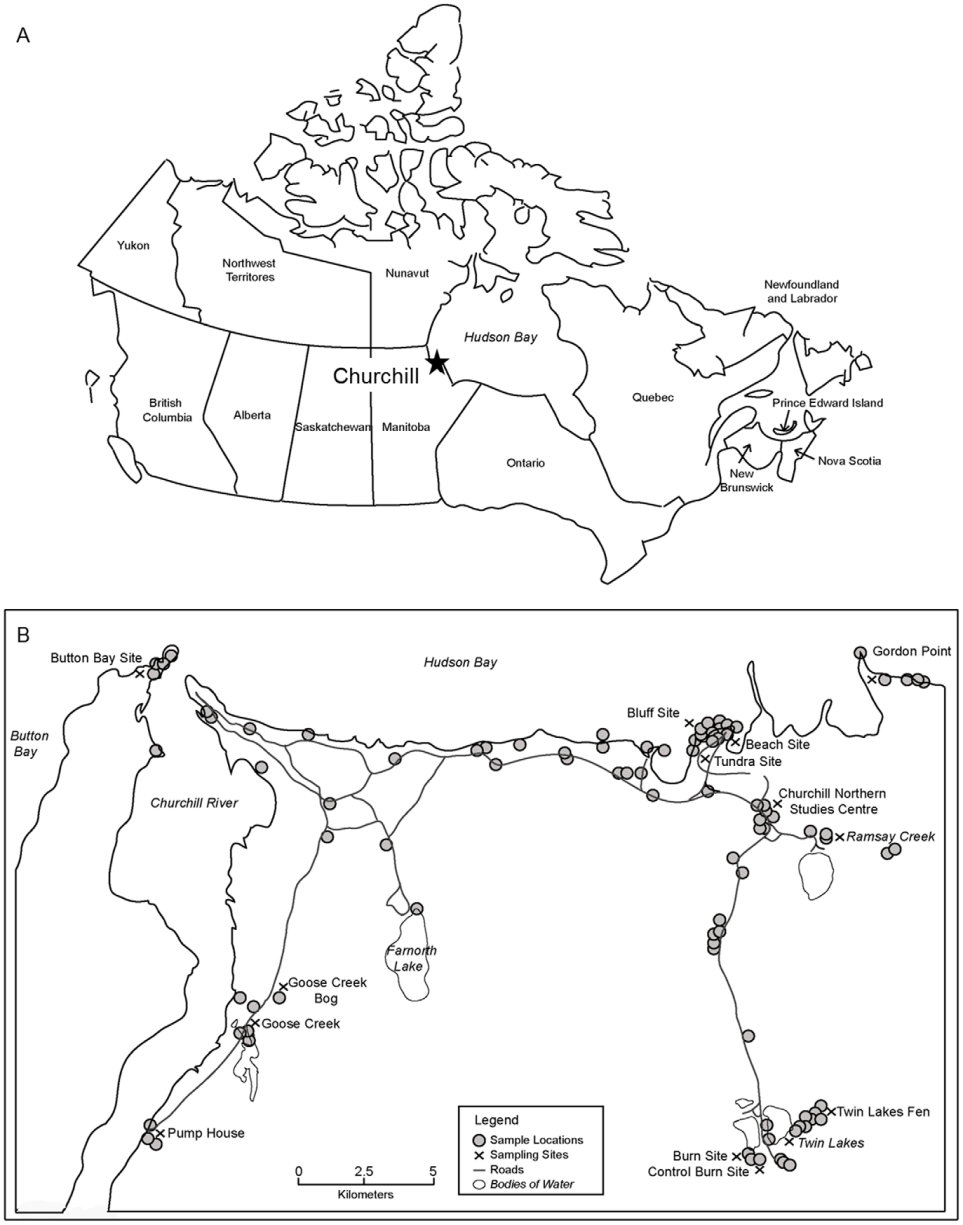
Sampling locations at Churchill. Maps depict a) the location of Churchill in Canada, and b) all sample locations along with specific sampling sites in the Churchill region.

Specimens were collected in the vicinity of Churchill during the snow-free season from June through August 2008 to 2011 by sweep netting, pitfall traps, Berlese funnel extractions and aspirators (Figure 1). A more regimented survey in 2010 included systematic sampling from 7 substrates at 10 locations over a 6 week period in boreal forest, bog, fen, tundra, marine beach, and rock bluff habitats. Seven substrates were sampled at each locale including moss, soil, litter, woody debris and lichens (*Cladina spp., Peltigera leucophlebia, Parmelia/Hypogymnia).* Approximately 500 mL of material from each substrate was collected, and extracted using modified Berlese funnels into 95% ethanol (EtOH). As well, two transects of five pitfall traps (with 95% EtOH) were deployed at each site and visited every 3 days for a total period of 9 days. Specimens were removed at each visit and placed into fresh 95% EtOH. Each of the pitfall transects and each substrate sample was treated as a separate analytical unit.

### Sorting/Identifications

The specimens in each analytical unit were sorted into morphospecies, and 3–5 specimens of each were selected for sequence analysis. In total 8240 specimens (approximately 14% of the total catch) were selected for analysis. All specimens were identified to a family level using keys in Krantz and Walter [16], and sarcoptiform mites were identified to genus.

### Barcoding Methodology

Each specimen was photographed and subsequently placed in a well containing 50 µl of 95% EtOH in a 96 well microplate. Collection details for each specimen together with its taxonomic assignment and its photograph are provided in a single data set on BOLD. The records can be retrieved using a DOI (http://dx.doi.org/10.5883/DATASET-MTBAR12N), a novel feature on BOLD enabling easy access and citability of barcode data [33]. Specimens were sequenced for the barcode region of the COI gene using standard protocols at the Canadian Centre for DNA Barcoding (CCDB) [34], using a cocktail of LepF1/LepRI [5] and LCO1490/HCO2198 [35] primers. Failed amplification reactions were further processed using the MLepF1 (Hebert unpublished) and MLepR2 (Prosser unpublished) primers. Glass fibre extraction was employed followed by voucher recovery [36]. DNA extracts were placed in archival storage at −80°C at the Biodiversity Institute of Ontario (BIO). Vouchered specimens were stored in 95% EtOH or slide mounted in Canada balsam, and deposited at both BIO and the Canadian National Collection of Insects, Arachnids and Nematodes.

Contigs were assembled and edited using CodonCode Aligner v. 3.0.1, and aligned by eye in MEGA 5.03 [37]. Each sequence with a length greater than 500 base pairs (bp) and with less than 1% ambiguous sites (Ns) was assigned a Barcode Index Number (BIN) by BOLD (Ratnasingham and Hebert 2012, in prep). All sequences with a length of 300 bp or longer, and with less than 1% Ns were also assigned to MOTUs using jMOTU [38] with a threshold of 15 nucleotide changes (2.3%), which is generally consistent with BIN assignments by BOLD. These MOTUs were used for further analysis, and are referred to interchangeably as BINs.

All sequence records together with trace files and images are available on BOLD as a single citable dataset (http://dx.doi.org/10.5883/DATASET-MTBAR12N). The sequences are also available on GenBank (Accessions GC680425–GU680432, GU680434–GU680497, GU702808–GU702809, HM405807–HM405810, HM405830–HM405857, HM431992–HM431993, HM431995–HM431998, HM904908, HM907069–HM907086, HM907088–HM907127, HM907130–HM907134, HM907138–HM907180, HM907182–HM907327, HM907329–HM907484, HQ558324–HQ558388, HQ558390–HQ558476, HQ558478–HQ558511, HQ558513–HQ558537, HQ558539–HQ558542, HQ558544–HQ558611, HQ558613–HQ558625, HQ558627, HQ558629–HQ558669, HQ558671–HQ558720, HQ558722–HQ558791, HQ941470–HQ941573, HQ941576–HQ941579, HQ966220–HQ966228, HQ966230–HQ966236, HQ966238–HQ966247, JX833624–JX838789).

### Assessing Richness

We constructed specimen-based accumulation curves using random sampling and 1000 iterations for overall diversity, for each order, and for each family with more than 100 specimens, or with more than 10 BINs. This was done to assess diversity, and to determine which groups were undersampled. The slope of the accumulation curve for the last 10 specimens on the curve was calculated for each order, family, and others (families with fewer than 100 specimens or 10 BINs were pooled) to assess the completeness of sampling [39]. Clades with a slope >0.1 were viewed as very undersampled, while those with a slope >0.01 indicated lineages with modest undersampling. Predictions of total mite richness and richness of each order were also calculated using specimen-based Chao’s species richness estimator [40] using the vegan package in R [41], [42].

### Faunal Similarity

Faunal similarity was assessed for samples collected systematically between the previously outlined sites and substrates. The similarity in community composition was visualized using cluster dendrograms computed from complete linkage hierarchial clustering method on Hellinger transformed abundances [43] and Bray Curtis dissimilarities using the vegan package in R [41], [42]. To test the significance of the clustering pattern between site type (forested or non-forested), we conducted an analysis of similarity (ANOSIM) with 999 permutations using the vegan package in R [41], [42].

## Results

### Assessing Richness

Barcode sequences were recovered from 6365 of the 8240 specimens, a success rate of 77.2% (Table 1). However, there was significant variation (*x* ^2^_2_ = 60.7, p<0.001) in recovery success among the three orders with a high of 80.4% for Sarcoptiformes and a low of 68.2% for Trombidiformes (Table 1). Most sequences (98.6%) were greater than 300 bp in length with less than 1% Ns. There were representatives of 899 BINs, with an average of 6.9 specimens per BIN. Mesostigmata was the least diverse order, accounting for 15% of the total diversity, while Sarcoptiformes composed 36% of the total diversity (Table 1). Trombidiformes were the most diverse order with 437 BINs (Table 1), accounting for 49% of the total diversity.

**Table 1:** Observed and expected BIN richness for each order, as calculated by Chao’s estimator in R including error (±SE) estimates.

Taxon	SequencingSuccess (%)	BINs	n	Chao	Slope of Accumulation Curve	# of Families
Mesostigmata	76.5	135	849	173 (±16)	0.049	17
Sarcoptiformes	80.4	327	3497	423 (±27)	0.022	39
Trombidiformes	68.2	437	1933	633 (±38)	0.093	21
Total	77.2	897	6279	1229 (±49)	0.050	77

**Figure 2:**
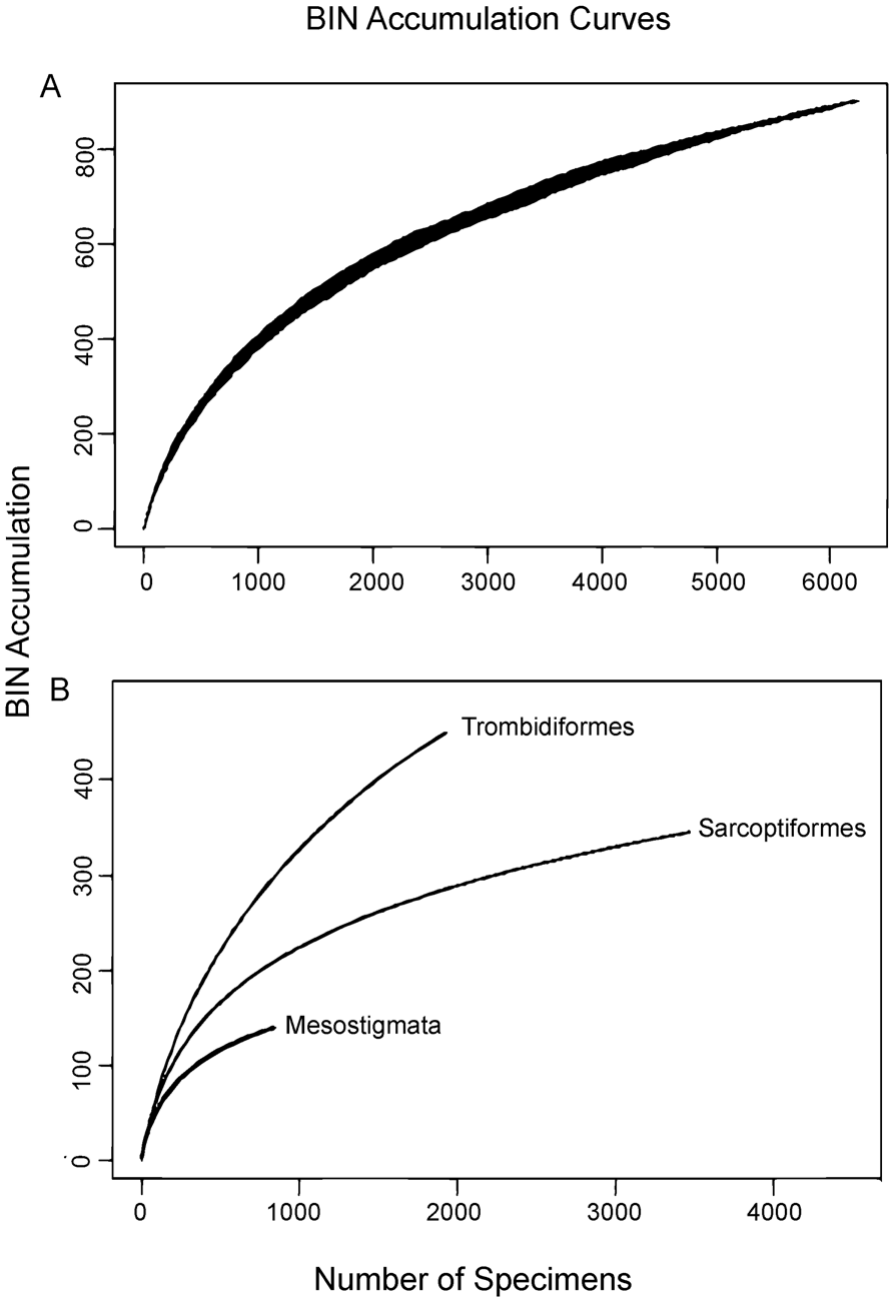
Bin accumulation curves. Curves represent a) the overall dataset, with 899 BINs from 6279 samples, and b) curves for each order - Trombidiformes (437 BINs, n = 1933), Mesostigmata (135 BINs, n = 849), and Sarcoptiformes (327 BINs, n = 3497).

Although the overall BIN accumulation curve did not reach an asymptote (Figure 2), there was sufficient data to estimate total mite diversity at Churchill as 1229 (±49) BINs (Table 1). The Sarcoptiformes was the best sampled order with a final accumulation curve slope of 0.022, and an estimated BIN richness of 423 (±27), while Mesostigmata were moderately well sampled with a final accumulation curve slope of 0.049, and an estimated richness of 173 (±16) BINs. The Trombidiformes was the least well-sampled order with a final accumulation curve slope of 0.093 and an estimated BIN richness of 633 (±38) (Table 1).

Among the five Mesostigmata families which met the requirements for detailed analysis, only the Zerconidae had a terminal slope of <0.01 (Table 2). Two families, Phytoseiidae and Melicharidae, had a slope >0.1 indicating that they include a very high number of uncollected species. Eleven of the 30 families of Sarcoptiformes met the requirements for analysis and 7 had a terminal slope >0.01 (Table 2). Four families (Ceratozetidae, Haplozetidae, Mycobatidae, Trhypochthoniidae) were well sampled, while two (Suctobelbidae, Brachychthoniidae) had terminal slopes ≥0.1 (Table 2). Ten of the 21 families of Trombidiformes met the requirements for analysis and all were under-sampled (>0.01 slope) with six families exceeding the 0.1 slope (Table 2). The Scutacaridae and Siteroptidae exhibited very unsaturated accumulation curves, with slopes of 0.50 and 0.37 respectively (Table 2).

**Table 2:** Richness and terminal slope for the accumulation curve of selected families in three orders of Acari.

	Family	BIN #	n	Slope
Mesostigmata	Blattisociidae	18	93	0.074
	Laelapidae	17	137	0.042
	Melicharidae	12	29	0.200
	Phytoseiidae	22	90	0.111
	Zerconidae	7	129	0.007
	Others	47	326	0.043
Sarcoptiformes	Brachychthoniidae	65	246	0.130
	Camisiidae	21	228	0.023
	Ceratozetidae	31	466	0.007
	Haplozetidae	3	147	0.003
	Mycobatidae	8	264	0.000
	Oppiidae	33	324	0.034
	Nanorchestidae	14	88	0.075
	Scheloribatidae	13	134	0.012
	Suctobelbidae	10	40	0.103
	Tectocepheidae	37	270	0.028
	Trhypochthoniidae	5	207	0.000
	Others	88	1079	0.015
Trombidiformes	Bdellidae	33	290	0.036
	Cunaxidae	20	60	0.157
	Erythraeidae	17	113	0.050
	Eupodidae	78	518	0.045
	Rhagidiidae	53	213	0.078
	Scutacaridae	21	32	0.500
	Siteroptidae	24	43	0.366
	Stigmaeidae	33	119	0.127
	Tarsonemidae	15	33	0.199
	Tydeidae	40	186	0.081
	Others	54	136	0.219

All slopes except for Ceratozetidae, Haplozetidae, Mycobatidae, Trhypochthoniidae, and Zerconidae exceed 0.010.

The inclusion of qualitative samples from 2011 increased overall BIN richness by 30% (206 BINs), and increased the overall estimate of richness by 26% (253 BINs) (Table 3). Mite richness increased similarly among the orders, ranging from 28–36% (Table 3).

**Table 3:** Effect of sampling patchy, short-lived habitats.

Taxon	Additional BINs	% increase	Additional Chao’s projection	% increase
Mesostigmata	36	36	50	40
Sarcoptiformes	71	28	99	30
Trombidiformes	99	29	98	18
Total	206	30	253	26

### Faunal Similarity

**Figure 3:**
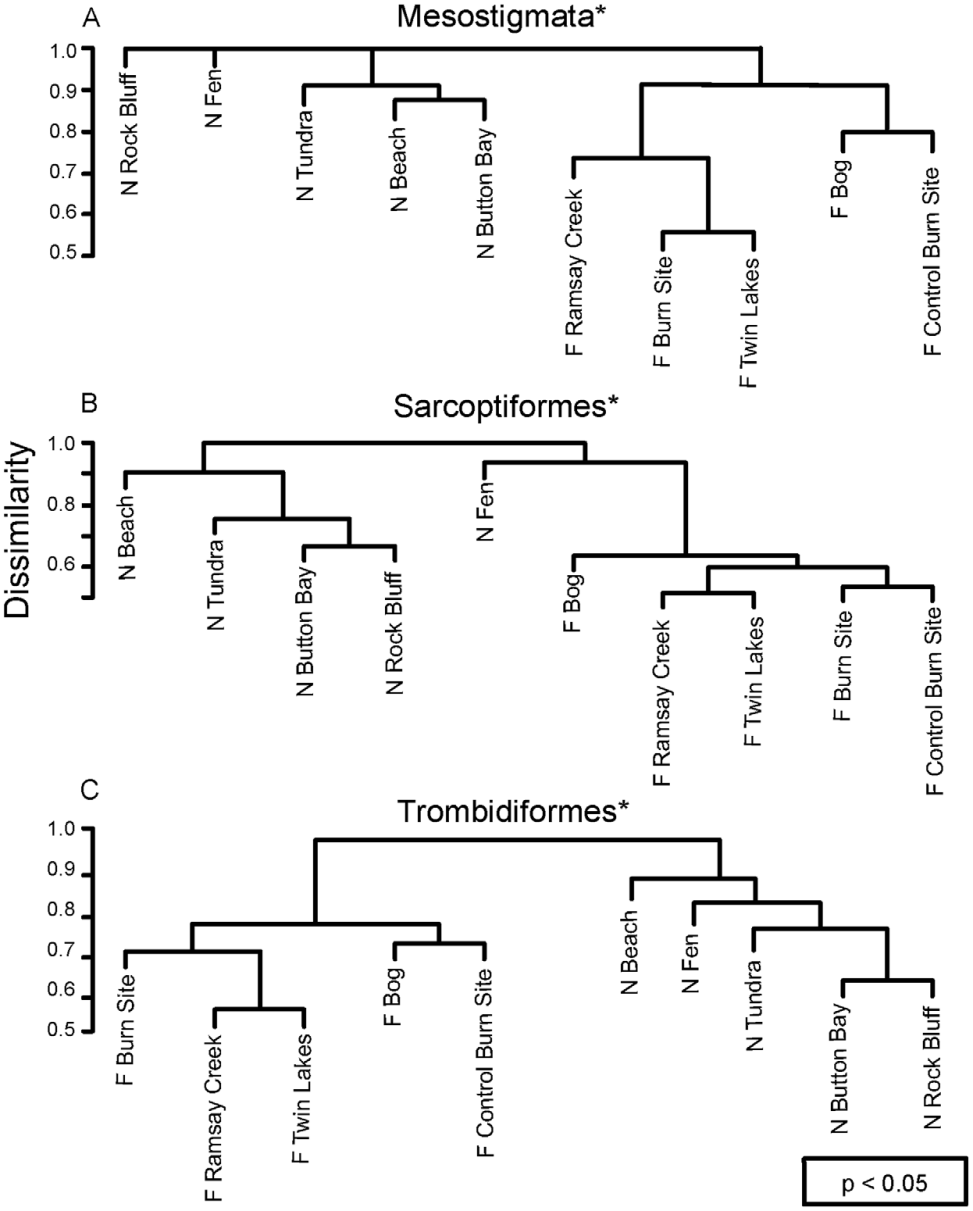
Cluster dendrograms showing the similarity of species assemblages for three mite orders among 10 sites in (F) and non-forested (N) settings.

**Figure 4:**
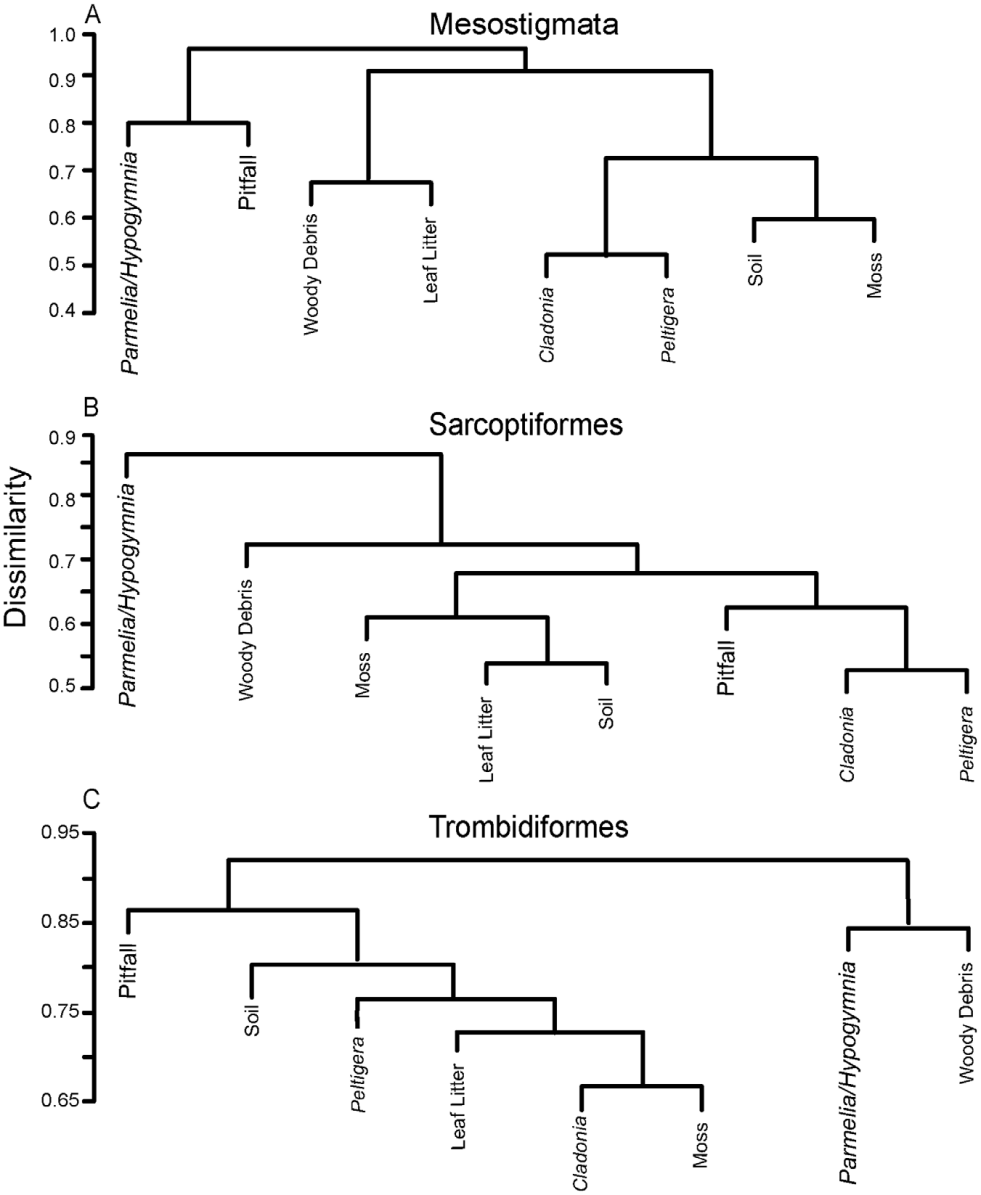
Cluster dendrograms of showing the similarity of species assemblages for three mite orders among 8 substrates.

Mites showed high turnover between samples from different sites and substrates, with mean Bray-Curtis Dissimilarities ranging from 0.73 to 0.93 among the three orders. All three orders showed similar clustering patterns among sites, with distinct separation between forested and non-forested sites (ANOSIM Mesostigmata R = 0.396, p = 0.01; Sarcoptiformes R = 0.616, p = 0.004; Trombidiformes R = 0.620, p = 0.004) (Figure 3). When looking at faunal similarity between substrates, slightly different patterns were revealed. In all three orders the fauna from the arboreal lichens (*Parmelia*, *Hypogymnia*) was very distinct, as well as the fauna from woody debris (Figure 4). However, the trombidiform and mesostigmatan faunas from pitfall traps were also highly dissimilar to those from other substrates, while the Sarcoptiformes from pitfall traps was similar to the fauna found on forest floor lichens such as *Peltigera* and *Cladonia* (Figure 4).

## Discussion

### Sequencing Success

Varied primer binding and size differences between the major groups of mites may be responsible for variation in sequence recovery. Most species of Trombidiformes, the order with the lowest success, are very small so DNA concentrations may have been too low for successful PCR amplification. Some Mesostigmata are more heavily sclerotized than the other lineages, perhaps also reducing DNA recovery. Okassa et al. [44] reported problems in recovery of Cyt B sequences in their work on phytoseiid mites when DNA concentrations were less than 2.33 ng/ul. A shift to smaller elution volumes might improve success by producing higher DNA concentrations. Homogenizing specimens should aid DNA recovery [45], but it would lead to the destruction of specimens preventing their subsequent taxonomic study. Designing and utilizing taxa specific primers might also increase amplification success [46], but primer design requires prior taxonomic knowledge and affiliated reference sequences, both of which are usually unavailable for mites.

### Assessing Richness

Our work has revealed the extreme diversity of the mite fauna at Churchill, despite our failure to collect and sequence all taxa. Sarcoptiform mites were better sampled than the other two orders as only 23% of the expected fauna remains undocumented. Lower sequencing success may at least partially account for lower completeness of species coverage for the other two orders. Sarcoptiform mites were the most abundant group in our samples, suggesting that our collection methods and scale of analysis were adequate to encounter most taxa. Alternatively, the Sarcoptiformes may include fewer rare species or show less local structure [47]. Mesostigmatan mites were moderately sampled, but 22% of the predicted fauna awaits collection. Their lesser coverage may be an artefact of their low abundance, a more patchy distribution, higher proportion of rare species [47], or lower success in sequencing. The accumulation curve of Trombidiformes is the least saturated, indicating 31% of trombidiform species await detection. With hyperdiverse groups increasing sample size will eliminate some of the singletons in the data but will invariably add new ones [48].

**Figure 5:**
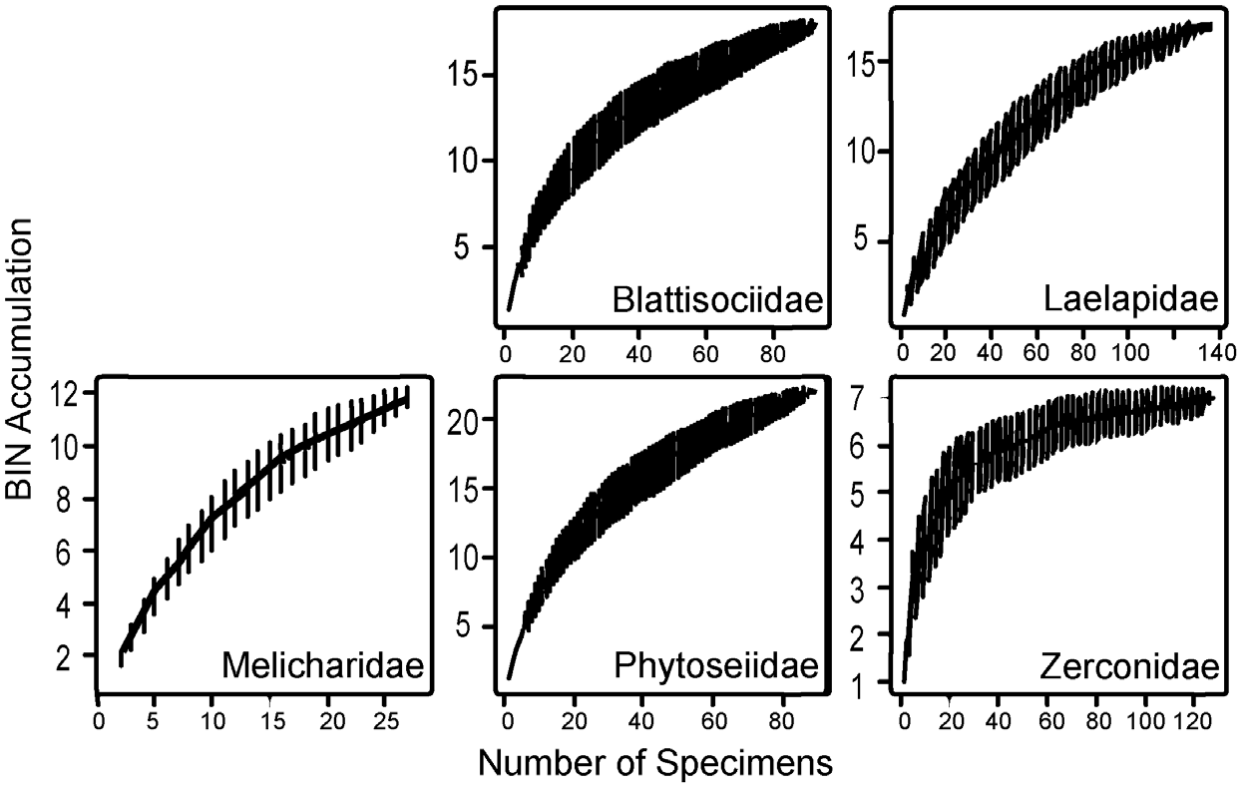
BIN accumulation curves for 5 families of Mesostigmata.

**Figure 6:**
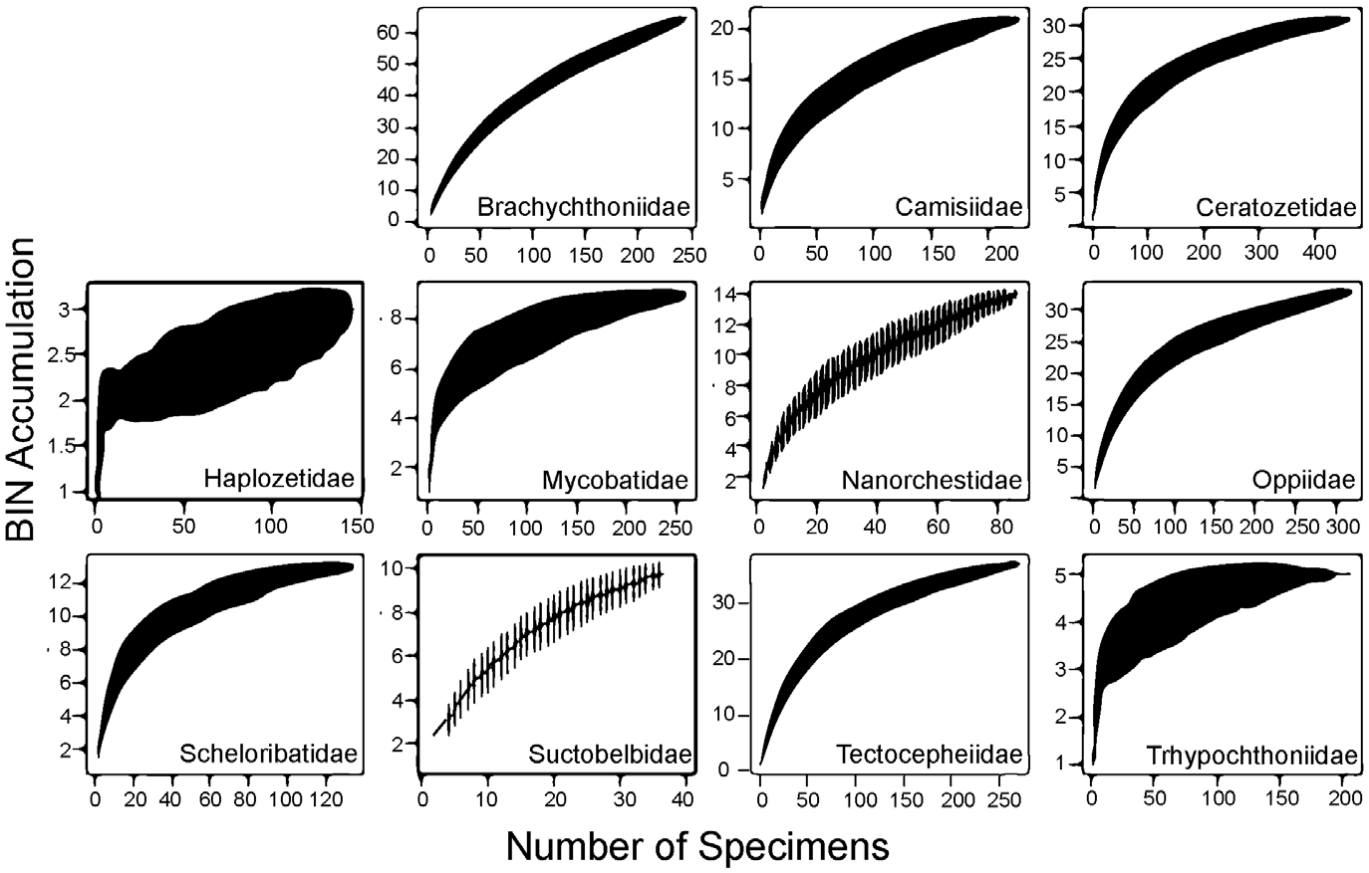
BIN accumulation curves for 11 families of Sarcoptiformes.

**Figure 7:**
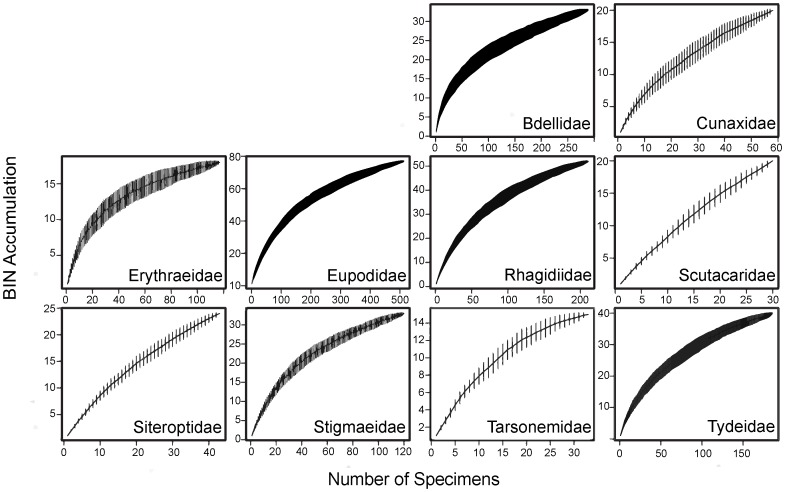
BIN accumulation curves for 10 families of Trombidiformes.

Similar trends in BIN richness and sampling saturation were also evident in the family accumulation curves (Figures 5, 6, 7) with most mesostigmatan families except the Zerconidae showing evidence of undersampling (Figure 5). Additionally, accumulation curves for all trombidiform families are unsaturated (Figure 7), implying general undersampling of this order. By contrast, all families of Sarcoptiformes except the Suctobelbidae and Brachychthoniidae showed a close approach to asymptotic diversity (Figure 6). However, differences in sampling saturation could be a result of differing sequencing success rates.

Despite incomplete sampling at Churchill, we found much greater acarine richness than recorded in past studies. Danks [49] reported 342 species of mites from the North American arctic, with 76 species of Mesostigmata, 144 Sarcoptiformes, and 122 Trombidiformes. We found nearly three times as many taxa at a single site, Churchill, with numbers increased by 78%, 127% and 258%, respectively from those reported by Danks [49]. Our sampling methods were likely to encounter a larger fraction of the species than the studies reviewed by Danks [49] which mainly assessed acarine diversity in soil cores. A few prior acarine surveys have generated species lists for entire countries [50], [51], but most are restricted to an order or family [52], [53]. Interestingly, the number of mite species which we detected using molecular methods at Churchill is close to the counts for New Zealand (1200 species [51]), and the UK (1700 species [50]).

No members of two other mite orders (Opiliocariformes, Holythrida) have been reported from the arctic [49] and they were absent from our samples. Their absence is unsurprising as both are small orders found in tropical/warm temperate climates. The Trombidiformes accounted for 49% of the species in our samples, a considerably higher level than 35% reported for the Canadian arctic [49], 23% for the Canadian subarctic [54], and 39% for the UK [50]. However, our results do conform with estimates from the high arctic (40% to 63% [54]), and with global acarine species descriptions as 48% are Trombidiformes [50]. Trombidiformes also compose 56% of the descriptions in North America, and 49% of the Australian fauna [50]. Trombidiformes have generally been thought to be a less important component of the acarine fauna in the subarctic, but our results challenge this conclusion. It is possible that insufficient efforts have been made to characterise the Trombidiformes of these regions, or that the resident species are more morphologically cryptic.

Sampling techniques can have an important impact on faunal discovery. Regimented sampling methods such as transects often overlook taxa that are rare or patchily distributed among sites. For example, a strict sampling technique revealed only 63% of the ant fauna known from La Selva [48]. More importantly, the species accumulation curve prematurely reached an asymptote, underestimating the true species richness [48]. Systematic sampling tends to capture dominant species, but often overlooks rare or transient species [48], [55]. By sampling patchy and temporary habitats, we dramatically increased the discovery of mite species at Churchill, most markedly in the Mesostigmata, reinforcing the notion that they are patchily distributed. The importance of microhabitats as potential refugia for rare species has been demonstrated for mesostigmatan mites, where most microhabitats contained only 2–3% of the collected species [56]. Behan-Pelletier [57] captured only one fifth of the fauna using systematic biodiversity sampling, while the rest of the fauna were uncovered by qualitative sampling of patchy habitats. This demonstrates that strict biodiversity surveys do not capture the complete fauna of a region, and emphasises the importance of qualitative sampling of ephemeral habitats to capture rare species.

### Faunal Similarity

We found marked divergence in the mite faunas from forested and tundra settings. Prior studies have established that the species composition of mite communities can be influenced by vegetation type [58]. However, it is generally thought that the composition of soil mite communities is more strongly correlated with soil moisture, although vegetation type typically covaries with moisture [28], [58]. Rouse [59] showed that amount and seasonal patterns of soil moisture in Churchill are significantly different between forest and tundra sites, variation which may explain the distinctness of their mite communities. One exception to this pattern was the fauna of fens which are wet, but treeless habitats. The sarcoptiform fauna from the fen grouped more closely with the forested sites, whereas the trombidiform fauna was more similar to the tundra sites. The Trombidiformes may have been less impacted by the high soil moisture of the fen as they tend to be active surface predators [16], while the Sarcoptiformes are less mobile and are influenced by heterogeneity in soil [60].

Mite faunal similarity patterns between substrates were less obvious than those between sites. Our single arboreal substrate samples did not allow for a clear comparison between arboreal and forest floor substrates such as described by Lindo and Winchester [61]. However, arboreal lichens (*Parmelia/Hypogymnia*), woody debris, and pitfall samples formed the most distinct communities. The pitfall traps likely catch a functionally different fauna [55], [62], such as the fast active predators moving across the soil surface, as well as those that may be phoretic on other insects caught in the trap. Woody debris is slightly more ephemeral in nature, perhaps providing a unique microhabitat for transient species or more specialized species. On the other hand, the arboreal lichens potentially represent a distinctly different faunal community living in arboreal substrates [61], and represent a largely undocumented source of acarine diversity in Churchill.

### Conclusion

Our study has revealed the power of DNA barcoding to provide insights into the diversity and distributional patterns of mites that could not have been gained through morphological approaches. Because of its use, we were able to analyze all life stages and both sexes, revealing a mite fauna with a species richness rivalling the most diverse of temperate habitats. Our work has also indicated the value of supplementing systematic sampling designs with qualitative sampling to ensure the examination of novel habitat types. The vouchered specimens generated through this study represent a valuable resource for future taxonomic research, particularly since specimens are partitioned into genetically cohesive assemblages. The utility of DNA barcoding for local biodiversity assessments is clear, but it will bring particular power to analyses which seek a deeper understanding of beta diversity patterns.
